# Propionic Acid Induces Gliosis and Neuro-inflammation through Modulation of PTEN/AKT Pathway in Autism Spectrum Disorder

**DOI:** 10.1038/s41598-019-45348-z

**Published:** 2019-06-19

**Authors:** Latifa S. Abdelli, Aseela Samsam, Saleh A. Naser

**Affiliations:** Burnett School of Biomedical Sciences, College of Medicine, University of Central Florida, Orlando, FL 32816 USA

**Keywords:** Crohn's disease, Autism spectrum disorders

## Abstract

Autism spectrum disorder (ASD) is a neurodevelopmental disorder characterized by glia over-proliferation, neuro-inflammation, perturbed neural circuitry, and gastrointestinal symptoms. The role of gut dys-biosis in ASD is intriguing and should be elucidated. We investigated the effect of Propionic acid (PPA), a short-chain fatty acid (SCFA) and a product of dys-biotic ASD gut, on human neural stem cells (hNSCs) proliferation, differentiation and inflammation. hNSCs proliferated to 66 neuropsheres when exposed to PPA *versus* 45 in control. The neurosphere diameter also increased at day 10 post PPA treatment to (Mean: 193.47 um ± SEM: 6.673 um) *versus* (154.16 um ± 9.95 um) in control, p < 0.001. Pre-treatment with β-HB, SCFA receptor inhibitor, hindered neurosphere expansion (p < 0.001). While hNSCs spontaneously differentiated to (48.38% ± 6.08%) neurons (Tubulin-IIIβ positive) and (46.63% ± 2.5%) glia (GFAP positive), PPA treatment drastically shifted differentiation to 80% GFAP cells (p < 0.05). Following 2 mM PPA exposure, *TNF-α* transcription increased 4.98 fold and the cytokine increased 3.29 fold compared to control (P < 0.001). Likewise, GPR41 (PPA receptor) and pro-survival p-Akt protein were elevated (p < 0.001). PTEN (Akt inhibitor) level decreased to (0.42 ug/ul ± 0.04 ug/ul) at 2 mM PPA compared to (0.83 ug/ul ± 0.09 ug/ul) in control (p < 0.001). PPA at 2 mM decreased neurite outgrowth to (80.70 um ± 5.5 um) compared to (194.93 um ± 19.7 um) in control. Clearly, the data supports a significant role for PPA in modulating hNSC patterning leading to gliosis, disturbed neuro-circuitry, and inflammatory response as seen in ASD.

## Introduction

Autism spectrum disorder (ASD) is a childhood onset, lifelong debilitating condition characterized by impaired social communication, stereotyped or repetitive behaviors, and spectrum-wide stages of mental retardation^1–3^. The prevalence of ASD in the United States has alarmingly increased in the last two decades. According to the Centers for Disease Control and Prevention (CDC), ASD rose from 1 out of 150 children in 2000, 1 in 68 in 2012, to a striking 1 in 59 children in 2018^3^. Currently, Management of ASD is limited to aggressive behavioral therapies focused on increasing independence and reducing major ASD characteristics^4^. Therefore, more research is urgently needed to understand the genetics and possible environmental cofactors, which may play a role in ASD development.

According to the Simmons Foundation Autism Research Initiative (SFARI) gene database, thousands of genes have been associated with ASD^5^. However, the effect of these genetic alternations in ASD individuals remain unknown. ASD is believed to be the result of an interplay between genetic predisposition, environmental insults, and maternal immune system abnormalities during the early gestational period ^2,6,7^. Emerging clinical studies suggest a possible role for gut dys-biosis in ASD development^6–8^. A shift in the microbiome in autistic individuals compared with that of neurotypical peers was reported^6–9^. This included elevated *Clostridia spp*., *Bacteriodetes*, and *Desulfovibrio spp*. in ASD^7–10^. Interestingly, these micro-organisms are known to be active fermenters of dietary carbohydrates and fibers leading to production of energy metabolism byproducts such as acetate (AC), propionate (PPA), and butyrate (BA)^8–12^. Propionate is seemingly the SCFAs most produced by ASD prevalent micro-organisms and is universally used as a preservative in processed food due to its anti-fungal characteristic^7–12^. PPA in the gut illustrates the interplay between the microbiome identity and dietary habits (nature *versus* nurture).

In circulation, PPA passes through the blood brain barrier to modulate multiple cell signaling processes including energy metabolism, neurotransmitter synthesis and release, and lipid metabolism^9^. Meanwhile, excessive PPA level might be toxic. In neonatal Propionic Acidemia (PA), Propionyl CoA Carboxylase (PCC), involved in the metabolism of amino and fatty acids, is not functional due to a mutation in one of the two genes that code for its Alpha and Beta subunits; PCCA and PCCB. As a result, PPA accumulates in the blood causing severe seizures, movement disorders, gastrointestinal issues, aloofness, and overall developmental delays^13^. Interestingly, PA and ASD share most of their core symptoms with multiple case studies reporting ASD as a comorbidity to PA^13–15^. Furthermore, high levels of PPA, but not BA, acetate, or other SCFAs, have been reported in the stools of ASD individuals; however, how PPA is involved in the development of ASD remains largely unknown^9,15^.

PPA is believed to cause systematic mitochondrial dysfunction (MD), as evidenced by increased free acyl-carnitine (cofactor used to transport long-chain and very-long-chain fatty-acids into the mitochondria) in rats exposed to PPA^16^. Interestingly, more than 30% of ASD patients were also reported to have MD, and elevations in carnitine-bound unprocessed long-chain and very-long-chain fatty-acids; thus providing further evidence for the association between PPA and ASD^15^. However, it remains unclear how MD and/or disturbed fatty acid metabolism may cause autistic phenotype.

Attempts to resume autistic-like behavior in rodents by exposure to PPA at different developmental stages have been reported^14,15^. For instance, intracerebroventricular delivery of PPA in rats resulted in increased IL-6, TNF-α, and interferon-γ cytokine levels, disturbed fatty acid metabolism, and marked microglia (neuro- inflammatory macrophages) over-proliferation^14^. Nonetheless, it remains unclear how PPA may affect the other neuronal cell types (Neurons and glia) particularly during the most sensitive stages of neural development.

Neural stem cells give rise to neuroepithelial progenitor cells (NPCs) which then differentiate into neuronal or glial cells^17^. Glial cells including oligodendrocytes and astrocytes, play a role in neurons development, connectivity, and protection^18–20^. During traumatic brain injury, reactive glial cells proliferate and release fibrillary acidic protein (GFAP) to inhibit damaged axonal regeneration causing gliosis^17,18^. Furthermore, glial and microglial cells release inflammatory cytokines to clean up damaged cells and toxins, therefore causing neuro-inflammation^18–20^. Some investigators regard gliosis as a protective process as it clears up damaged cells and blocks regrowth of damaged axons^19^. However, it is safe assuming that if gliosis occurs during the earliest stages of brain development it will greatly affect neural architecture and connectivity. In the ASD brain, disturbed neuronal circuitry with increased regional cell density in cortical, limbic, and cerebellar regions were reported^21–24^. In the meantime, glial cell count far exceeded that of neurons^23,24^. Nevertheless, it remains unclear if premature gliosis may play a role in ASD.

Concurring evidence suggests that ASD may stem from a disorder in glial cells^19–23^. Specifically, GFAP was shown to be highly expressed in the ASD brain compared to age matched healthy controls^20,25^. Other studies reported that anti-tumoral, pro-apoptotic Phosphatase and tensin homolog (PTEN), was elevated in autistic astroglial cells^26^. This suggests an over-proliferation of glial cells in ASD. *Wen Yi, et al.* reported that transgenic mice with astroglial specific deletion of PTEN, demonstrated altered radial glia cell proliferation and disturbed neuronal patterning^27^. Most intriguingly, reduced microbiota complexity was directly linked with impaired microglial proliferation and maturation in germ free mice^28^. In contrast, upon re-introduction of *Clostridium cluster XIV, Bacteroides distasonis, and Lactobacillus salivarius* strains to the GI tract, microglial phenotype was partially restored^28^. It was further established that such effect is facilitated by SCFA produced by these bacteria. Nevertheless, the paper does not provide cues on the proliferation state of the main brain cells; neurons and glia.

PPA interacts with brain cells *via* G-protein-coupled SCFA receptors including GPR41^29^. Since PPA (3 carbons) is the most potent activator of GPR41, and due to the prevalence of *Clostridia spp*., *Bacteriodetes*, and *Desulfovibrio spp*. in ASD microbiome and its association with elevated PPA and gliosis^30^, we hypothesize that elevated PPA may tamper with neural cell plasticity and differentiation *in vitro* leading to gliosis, increased inflammatory profile, and disturbed neural connectivity, similar to ASD.

## Materials and Methods

### Culture of hNSCs *in vitro*

StemPro Neural Stem Cells (Cryopreserved human fetal-derived neural stem cells (NSCs); Thermo Fisher Scientific, A15654), were cultured in T-25 suspension flask containing 15 mL of KnockOut™ DMEM⁄F-12 Basal Medium (BM), GlutaMAX-I and StemPro® neural supplements, Heparin (6 units/ml), and Ascorbic acid (200 uM). Initially, cells were cultured as neurospheres in FGF Basic (b-FGF) and EGF recombinant proteins (20 ng/ml) media until they reached 80% confluency. They were passaged up to three times using StemPro Accutase® cell dissociation reagent (ThermoFisher; Cat#A11105) and then re-plated for further proliferation.

### Treatment with PPA, BA and GPR41 inhibitor

NSCs were treated with sodium propionate (PPA), and sodium butyrate (BA) (Sigma) at 0.1 mM, 0.5 mM, 1 mM, and 2 mM final concentration. Control cells were treated with 1X PBS. Another set of NSCs were pre-treated for 24 h with 2 mM ketone body β-hydroxybutyrate (β-HB), a potent inhibitor of GPR41 receptor, prior to PPA and BA treatments^31^. All treatments were done in triplicates (n = 3). Cells were then incubated for up to 10 days to form neurospheres. For differentiation purposes, intact neurospheres from each set were further plated on Geltrex (Cat# A14133; Fisher Scientific) pre-coated 24 well plates or 8 well chambers for an additional 7 to 10 days in a humidified 37 °C and 5% CO_2_ incubator. Differentiation media consisted of the regular StemPro® complete media without growth factors.

(n = 3) repetitions per treatment setting were used for differentiation experiments.

### Neurosphere count and diameter measurements

Neurospheres were imaged using a digital camera (Amscope MU130, USB 2.0 DC 5V, 250 mA) mounted on an inverted tissue culture microscope (40X–800x Amscope, Japan) at 10x magnification every other day for 4 time points post plating (Days 2, 4, 8, and 10). The total number of neurospheres with a minimum diameter of 25 um were counted. The diameter of at least 15 Neurosphere per condition was measured using Amscope software version x64, 3.7.7303. The average diameter of neurospheres per condition was reported.

### Neurosphere plating, differentiation, and immunostaining

Pre-treated neurospheres were sub-plated on 8-well chambers pre-coated with 1:100 Geltrex diluted in DMEM/F-12 media. Duplicate repetitions per treatment setting were used for differentiation experiments. Cells were allowed to differentiate for 7 to 10 days and then washed and fixed with 10% formalin and 3% Triton-X for 10 min before incubation in 10% normal goat serum (NGS; Vector Laboratories) for 1 h. To determine the ratio of glial *versus* neural cells in SCFA treated NSCs, cells were double immune-stained for GFAP (Glial cell marker) and Tubulin-IIIβ (Neural cell marker) markers. Briefly, slides were incubated at 4 °C over night with mouse anti-human-GFAP antibody (1:20 in 10% NGS; Sigma; Cat# SAB4100002) or rabbit anti-human Tubulin-IIIβ antibody (1:50; in 10% NGS Sigma; Cat# T5076). Cells were next washed and incubated for 1 hr with goat anti-rabbit FITC (Tubulin-IIIβ) or goat anti-mouse TRITC (GFAP); Sigma] diluted in 1:50 in PBS. For detection of GPR41 expression on neuronal cells, anti-GFAP and Tubulin-IIIβ stained cells were double-stained with rabbit anti-FFAR3 (GPR41; 5 ug/ml, Abcam; Cat# ab103718). Antifade Vectashield medium containing 4′,6-diamino-2-phenylindole (DAPI; Vector Laboratories) was used to co-stain nuclei. Amscope IN480TC-FL-MF603 Fluorescent Microscope and Leica confocal microscopes were used to visualize and image GFAP and Tubulin-IIIβ positive cells as well as GFAP/GPR41 and Tubulin-IIIβ/GPR41 double positive cells. Multiple channels were merged using ImageJ 1.39o software.

### Neurite outgrowth measurements

To evaluate the effect of PPA on neurite outgrowth, three 25X fluorescent images of Tubulin-IIIβ positive neurons were obtained using Amscope IN480TC-FL-MF603 fluorescent microscope mounted with an MF603C-CCD digital camera. Each experiment was repeated 3 times for accuracy and neurite outgrowth length (µm) from 15 random neurons per treatment setting were measured using Amscope software version x64, 3.7.7303. and averaged.

### Evaluation of gene expression using RT-PCR

Cells were plated in 24-well plates as described earlier and allowed to differentiate for 7 days followed by RNA extraction and cDNA synthesis.

#### RNA isolation

Total RNA was extracted using the TRI® reagent (Invitrogen) in RNase free environment. RNA was then separated from the aqueous phase in chloroform and precipitated in isopropyl alcohol followed by a washing step in 75% ethanol. Dried RNA pellets were suspended in TE buffer or RNAse free water and saved in −20 °C.

#### cDNA synthesis

cDNA was synthesized from 800 ng total RNA in 4 ul of iScript reverse transcriptase Supermix (Bio-Rad). Reverse transcription was carried out in MyGene Series Peltier Thermal Cycler under the following conditions: 5 min at 25 °C, 20 min at 46 °C and 1 min at 95 °C.

#### RT-PCR

The RT-PCR protocol included a 96-well Microamp RT-PCR reaction plate and the 7500 Fast Real-Time PCR System (Applied Biosystems). The 20 ul reaction mixture contained 1 µL of cDNA (30 ng/µL), 10 µL of Fast SYBR Green Mastermix (Thermo Fisher Scientific), 1 µL of either IL-10, TNF-α, GFAP, Tubulin-IIIβ, Akt, PTEN, or GPR41 PrimePCR SYBR Green Assay mixes (Bio-Rad), and 8 µL of sterile water. Housekeeping GAPDH primer assay from Biorad was used to measure the endogenous baseline CT values. All experiments were repeated up to three times and each sample ran in duplicate. Relative mRNA expression levels were calculated by using the equation 2^(−∆CT)^, where ∆CT = [(Sample RT-PCR CT value) − (GAPDH CT baseline value)].

### Measurement of protein and cytokine levels

Cells were plated in 24-well plates as described earlier and allowed to differentiate for 7 days. Next, they were harvested by centrifugation at 300 RPM for 5 min and supernatant saved for cytokine analysis. To extract cellular proteins, cell pellets were incubated for 15 min in shilled RIPA lysis buffer (Thermo-Fisher; Cat# 89901), and then centrifuged at 14,000 RCF for 10 min. Supernatant containing protein homogenates were subjected to commercially available ELISAs specific to GFAP (LS-Bio; Cat# LS-F11533), Tubulin-IIIβ (LS-Bio; Cat# LS-F12697), GPR41 (LS-Bio; Cat# LS-F9213), PTEN (Cell Signaling; Cat# 7882), p-AKT (Cell Signaling; Cat# 7252), IL-10 (Sigma-Aldrich; Cat# KHC0101) and TNF-α (LS-Bio; Cat# EH3TNFA) as described by the manufacturer. All ELISA experiments were ran in duplicate (n = 2) with each sample loaded in triplicate and averaged.

### Statistical analysis

Statistical analysis was performed using GraphPad Prism 7.02 software. Significance among experiments was assessed by either Unpaired Two-tailed t test or one-way analysis of variance (ANOVA) followed by Tukey’s multiple comparison test and cross checked with Wilcoxon matched-pairs test for non-parametric tests (# of (n) repetitions superior to 5 and inferior to 20; 5 < n < 20). Data is presented as (Mean ± Standard Error of the Mean (SEM)). Statistical significance is assigned for p-value < 0.05 and confidence interval exceeding 95%. P-values < 0.001, and p-values < 0.0001 are also mentioned when achieved. F-Statistic value is presented as F (DFn, DFd).

## Results

### PPA and BA enhance neural stem cell proliferation *in vitro*

**Figure 1:**
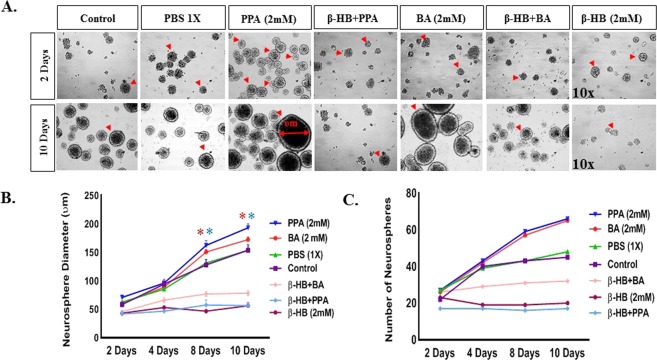
SCFAs Enhance Neural Stem Cell Proliferation *In Vitro*. Panel A depicts 10x representative bright field representative images of neurospheres at day 2 and 10 for the following treatments; Control, PBS (1X), PPA (2 mM), β-HB + PPA, BA (2 mM), β-HB + BA, and β-HB alone. Red arrows are pointing towards neurospheres. Graph 1B represents quantitative data for neurosphere diameter (um) averages per treatment group over day 2, 4, 8, and 10. Graph 1C depicts number of neurospheres averages per treatment group over day 2, 4, 8, and 10. Data is represented as Mean + SEM (n > 10 neurospheres per group and time setting) and statistical significance: *p < 0.0001, F (6, 286) = 48.71 (Wilcoxon matched-pairs and One-way ANOVA followed by Tukey’s post-hoc test). Red and blue (*) represent significance of the corresponding color treatment vs. its Control.

To understand the effect of PPA on hNSCs proliferation *in vitro*, we treated the cells with PPA (2 mM), BA (2 mM), or PBS (1x) and monitored neurosphere formation for 10 days. As shown in Fig. 1A, at day 2 following treatments, neurosphere diameter averaged (Mean: 58.45 um ± SEM: 4.1 um) across all treatments and the diameter differentially increased by day 10. When cells were pre-treated with 2 mM of β-HB (GPR41 inhibitor) before exposure to PPA or BA, the neurosphere diameter on day 10 averaged (56.53 um ± 4.74 um) for β-HB + PPA, (78.41 um ± 4.51 um) for β-HB + BA, and (56.26 um ± 5.73 um) for β-HB alone. Figure 1B illustrates the progress of the neurosphere expansion at 4 intervals within 10 days treatment. In absence of β-HB pre-treatment, PPA and BA increased neurosphere diameter significantly when compared to untreated cells (One-way ANOVA followed by Tukey’s multiple comparison test. p < 0.0001, F (6, 286) = 48.71). We also measured the neurosphere counts following each treatment (Fig. 1C). At Day 2, the neurospheres average count was (24 ± 3) for all treatments. At day 10, the neurospheres average count increased to 66 and 65 for PPA and BA, respectively. Cells treated with PBS had an average of 48 neurospheres. Cells pre-treated with β-HB contained 17, 32, and 20 total neurospheres after 10 days of treatment with PPA, BA, and β-HB alone, respectively.

### PPA promotes glial cells differentiation *in vitro*

**Figure 2:**
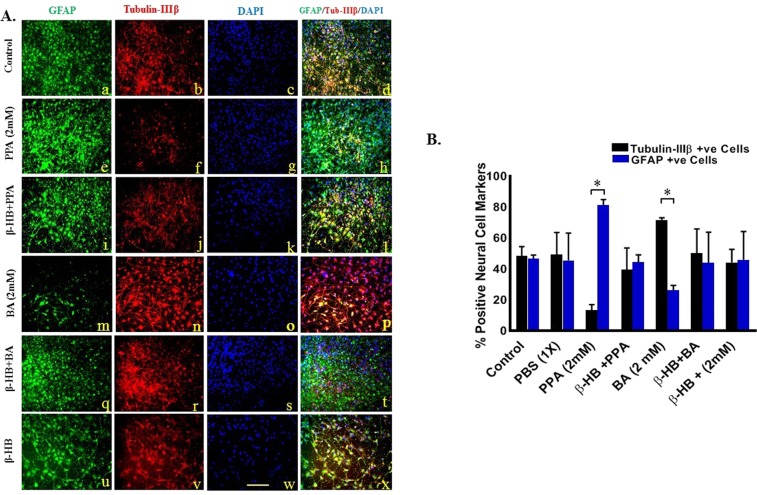
PPA Promotes Glial Cells Differentiation *In Vitro*. (**A)** Depicts fluorescent representative images of differentiated cells from hNSCs with green color for GFAP positive cells (a,e,I,m,q, and u), red for Tubulin-IIIβ positive cells (b,f,j,n,r, and v), blue color for DAPI positive cells (c,g,k,o,s, and w), and merged images of all three channels per treatment in (d,h,l,p,t, and x) for Control, PPA (2 mM), β-HB + PPA, BA (2 mM), β-HB + BA, and β-HB treatments, respectively. Magnification 25x and scale bar 25 um. (**B)** Represents quantitative analysis of the Mean of % positive neural cell markers over total DAPI with black bars for Tubulin-IIIβ positive, and blue bars for GFAP positive cells. Data was collected from a minimum of (n = 3) random areas per duplicated treatment setting. Statistical significance was tested using One-way ANOVA followed by Tukey and confirmed with Wilcoxon matched-pairs tests. (*p < 0.05, F (13, 28) = 2.520), for GFAP vs Tubulin-IIIβ positive cells within the ([) limited treatments.

The effect of PPA and BA on hNSCs differentiation was evaluated by quantifying GFAP *versus* Tubulin-IIIβ positive immunostained cells in three different areas from each treatment. Figure 2A depicts confocal representative images of differentiated cells from hNSCs with blue color for DAPI positive cells, green for GFAP positive, and red for Tubulin-IIIβ positive cells, scale bar 25 um. As shown in Fig. 2B, *in vitro* plated untreated hNSCs have spontaneously differentiated to equal number of neurons and glia with (48.38% ± 6.08%) neurons and (46.53% ± 2.5%) glia (p < 0.05). There was no change when cells were treated with 1x PBS. Surprisingly, treatment with PPA drastically shifted hNSCs differentiation to more than 80% glia whereas Tubulin-IIIβ positive cells were only 13.20% of the total number of cells. On the contrary, BA shifted hNSCs differentiation to (77.78% ± 1.29%) neurons whereas GFAP positive cells drastically decreased (17.26% ± 3.23%). Interestingly, pre-treatment of hNSCs with β-HB resulted in cell differentiation similar to control cells (p < 0.05, Fig. 2B). Data was collected from a minimum of (n = 3) random areas per duplicated treatment setting. Statistical significance was tested using One-way ANOVA followed by Tukey multiple comparison test and confirmed with Wilcoxon matched-pairs tests. (*p < 0.05, F (13, 28) = 2.520), for GFAP vs Tubulin-IIIβ positive cells within the ([) limited treatments.

### Effect of PPA on *tubulin-IIIβ* and *GFAP*

**Figure 3:**
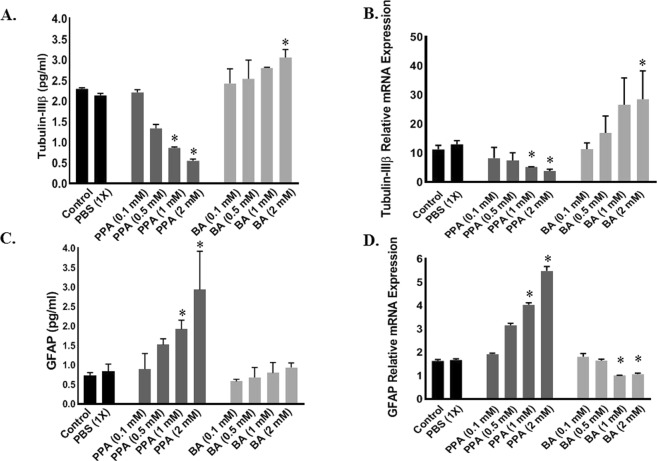
Effect of PPA on Neural *versus* Glial Cell Markers. Depicts; ELISA (**A**,**C**) and RT-PCR (**B**,**D**) analysis for Tubulin-IIIβ (**A**,**B**) and GFAP (**C**,**D**) under ascending concentrations of PPA and BA (0.1, 0.5, 1, and 2 mM). Black bars represent the controls (no treatment other than media) and media supplemented with 1x PBS. Data is represented as Mean + SEM (n = 3 per group) and statistical significance (*p < 0.0001 for Tubulin-IIIβ and p < 0.05 for GFAP) was obtained using either (Two-tailed Unpaired t test, Wilcoxon matched-pairs, and/or One-way ANOVA followed by Tukey’s post-hoc test) vs. Controls.

We measured protein and gene expression of both Tubulin-IIIβ and GFAP in each cell group. As shown in Fig. 3A, Tubulin-IIIβ protein level gradually decreased up to 5X following treatment with 2 mM PPA (*p < 0.0001, F (9, 10) = 17.91). Similarly, *Tubulin-IIIβ* relative mRNA expression significantly decreased after PPA treatment (Two-tailed Unpaired t test, p < 0.05, Fig. 3B). On the other hand, GFAP protein level and gene expression increased significantly following PPA treatment. Statistical significance was ran using One-Way ANOVA followed by Tukey’s multiple comparison tests (Protein *P < 0.05, F (9, 10) = 3.890 and gene expression p < 0.0001, F (9, 10) = 253.0), Fig. 3C,D.

An opposite effect on Tubulin-IIIβ and GFAP was observed in cells treated with BA. Figure 3A,B illustrates the positive increase in Tubulin-IIIβ protein and gene expression in cells treated with BA compared to significant decrease in GFAP protein and gene expression in these cells (*p < 0.05 Fig. 3C,D).

### Expression pattern of GPR41 on neurons *versus* glial cells

**Figure 4:**
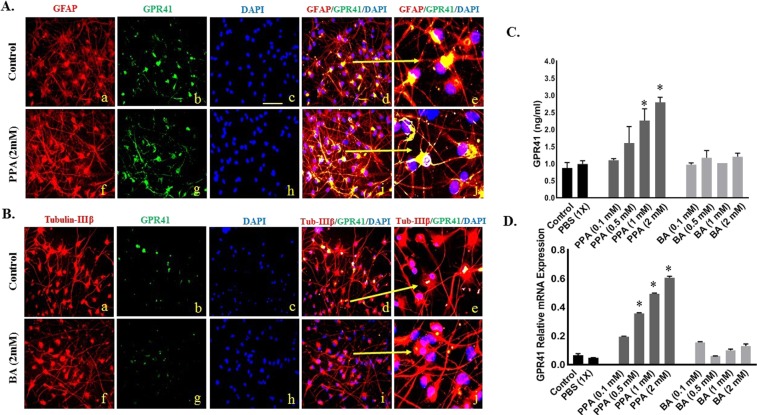
GPR41 Expression Pattern on Neurons *versus* Glial Cells. Panel A and B depicts double-immunostained representative images of differentiated cells double positive for GFAP + GPR41 (panel A) and Tubulin-IIIβ + GPR41 (panel B). GFAP and Tubulin-IIIβ stainings are depicted in red (a,f) in panels A and B, respectively. GPR41 is depicted in green (**B**,**C**), and DAPI in blue (**C**,**H**). Merged a + b + c channels are depicted in (d) and enlarged in (e) for control merged f + g + h channels in (i) and enlarged in (j) for PPA 2 mM treated cells (Panel A and BA treated cells for panel B). Magnification 25x and scale bar 25 um. (**C**,**D**) Represent ELISA and RT-PCR results for GPR41 expression under ascending concentrations of PPA and BA (0.1, 0.5, 1, and 2 mM). Black bars present the controls (no treatment other than media) and media supplemented with 1x PBS. Data is represented as Mean + SEM (n = 3 replicates per group) and statistical significance (*P < 0.0001, F (9, 10) = 718.4) was calculated using Wilcoxon matched-pairs and One-way ANOVA followed by Tukey’s post-hoc test vs. Controls.

To study the effect of PPA and BA on expression of GPR41 in differentiated hNSCs, we measured GPR41 protein level and relative mRNA expression. Figure 4A,B show confocal images representative of colocalization of GPR41 receptor on GFAP positive cells (Panel 4A) and Tubulin-IIIβ (Panel 4B). As shown in Fig. 4A, GFAP strongly co-localized with GPR41 in cells treated with PPA (image A-i and enlarged in image A-j) compared to untreated cells (image A-d and enlarged in image A-e). There was minimum co-localization between Tubulin-IIIβ and GPR41 following BA treatment (images B-d and B-i). Of note, although all groups were immune-stained and analyzed, we only chose to depict PPA and BA treatments along with their controls for accurate representation of the co-localization.

We also measured GPR41 protein level and relative mRNA expression following treatment with PPA and BA. As shown in Fig. 4C, PPA increased GPR41 protein at least 3X following treatment with 2 mM PPA (*P < 0.01, F (9, 10) = 8.66). Similarly, GPR41 relative gene expression increased several folds following PPA treatment (Fig. 3D, p < 0.05). There was no change on GPR41 level for cells treated with BA or PBS Fig. 4D). Statistical significance was ran using One-way ANOVA followed by Tukey’s multiple comparison tests (*P < 0.0001, F (9, 10) = 718.4).

### PPA Induces GPR41-mediated p-Akt survival pathway in differentiating glial cells

**Figure 5:**
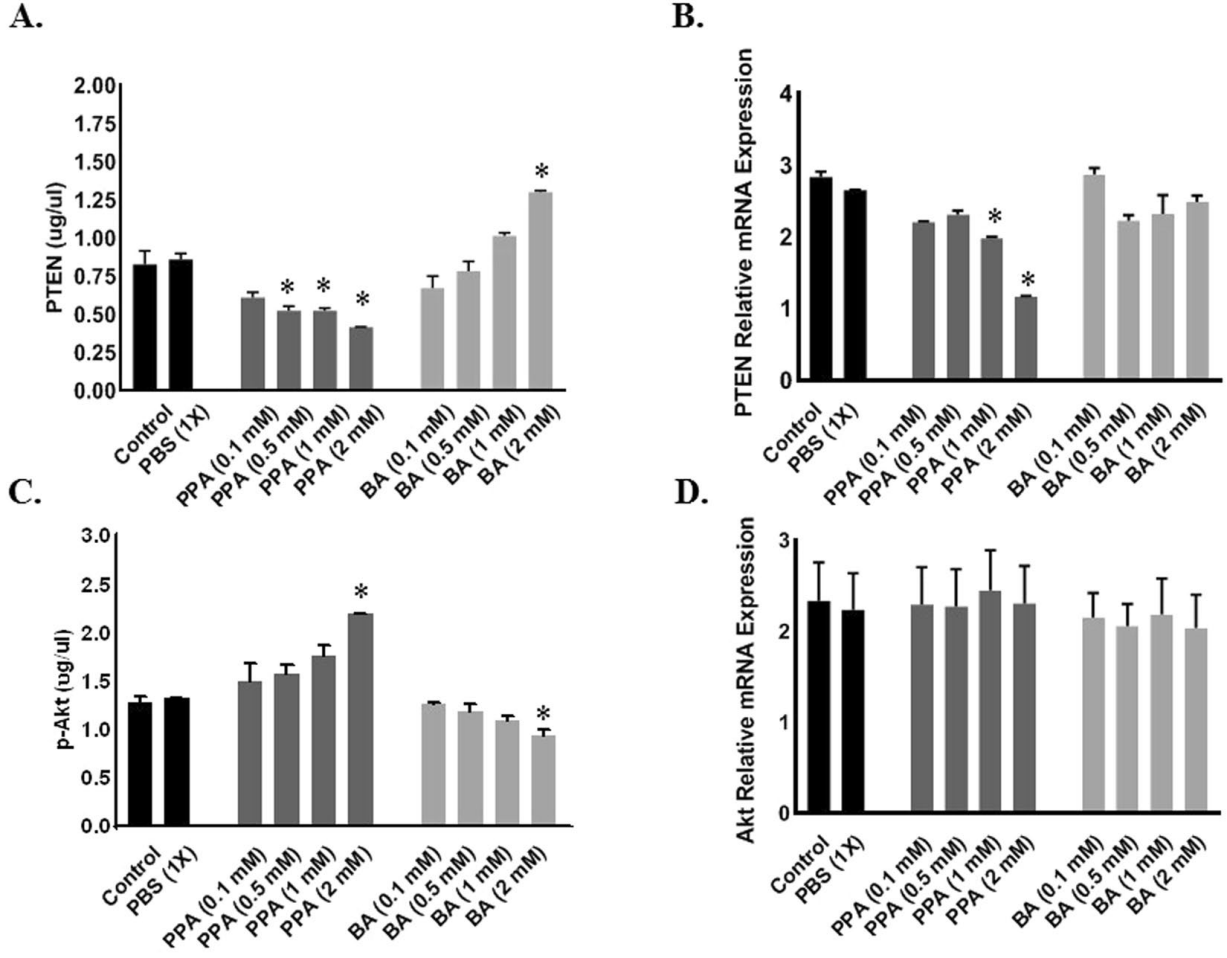
PPA Induces GPR41-Mediated p-Akt Survival Pathway in Differentiating Glial Cells. Depicts; ELISA (**A**,**C**) and RT-PCR (**B**,**D**) analysis for PTEN (**A**,**B**) and p-Akt/Akt (**C**,**D**) under ascending concentrations of PPA and BA (0.1, 0.5, 1, and 2 mM). Black bars represent the controls (no treatment other than media) and media supplemented with 1x PBS. Data is represented as Mean + SEM (n = 3 per group) and statistical significance: (*p < 0.001 for PTEN and p < 0.05 for p-Akt) was calculated using One-way ANOVA followed by Tukey’s post-hoc tests vs. Controls.

ELISA and RT-PCR were used to determine the *in vitro* effect of PPA and BA on expression levels of pro-survival Akt and its direct inheritor PTEN. As shown in Fig. 5, only 1.0 mM PPA was needed to significantly decrease PTEN both protein (A) and gene expression levels (B) compared to untreated cells (p < 0.0001). Although there was no change in *PTEN* expression following BA treatment (Fig. 5B), PTEN protein level increased significantly and seems to be dose-dependent in BA-treated cells (Fig. 5A) compared to controls (p < 0.0001 was achieved at 2 mM BA treatment, F (9, 10) = 49.86).

Similarly, ELISA results for the activated form of Akt (p-Akt) showed that ascending concentrations of PPA promotes an increase in available p-Akt to reach significance against control at 2 mM PPA (Fig. 5C). BA on the other hand achieved the exact opposite with a significant decrease in available p-Akt to reach the lowest significant (P < 0.0001, F (9, 10) = 18.42) level at 2 mM BA (Fig. 5C). Interestingly, there was no effect on *Akt* gene expression following any of the SCFAs treatments (Fig. 5D). Statistical significance was ran using One-way ANOVA followed by Tukey’s multiple comparison test.

### PPA promotes gliosis and Pro-Inflammatory cytokines release

**Figure 6:**
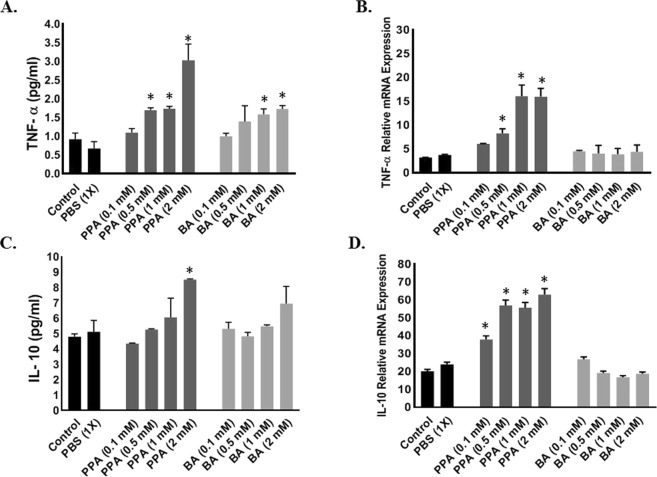
PPA-Induced Glial Activation, Promotes Inflammation. Depicts; ELISA (**A**,**C**) and RT-PCR (**B**,**D**) analysis for pro-inflammatory TNF-α (**A**,**B**) and Anti-inflammatory IL-10 (**C**,**D**) under ascending concentrations of PPA and BA (0.1, 0.5, 1, and 2 mM). Black bars represent the controls (no treatment other than media) and media supplemented with 1x PBS. Data is represented as Mean + SEM (n = 3 per group) and statistical significance (*p < 0.001 for TNF-α and p < 0.05 for IL-10) was calculated using One-way ANOVA followed by Tukey’s post-hoc tests vs. Controls.

To determine the function of PPA-induced Glia cell differentiation, we measured TNF-α and IL-10 released into the cell culture media and their corresponding mRNA in cells lysates. As shown in Fig. 6, TNF-α increased significantly at both the protein (P < 0.001, F (9, 10) = 9.174) and RNA (P < 0.0001, F (9, 10) = 15.72) levels when hNSCs were treated with a minimum of 0.5 mM PPA (Fig. 6A,B, respectively). Similarly, the anti-inflammatory IL-10 levels increased under ascending concentrations of PPA (Fig. 6C: P < 0.02, F (9, 10) = 4.147, and Fig. 6D: P < 0.0001, F (9, 10) = 81.23). Overall, TNF-α level has super exceeded the increase in IL-10 level leading to a net increase in the pro-inflammatory TNF-α. Although treatment with BA seems to increase TNF-α protein level, there was minimum change in TNF-α or IL-10 gene expression (Fig. 6). Statistical significance was ran using One-way ANOVA followed by Tukey’s multiple comparison test.

### The bivalent role of SCFAs on neurite growth *in vitro*

**Figure 7:**
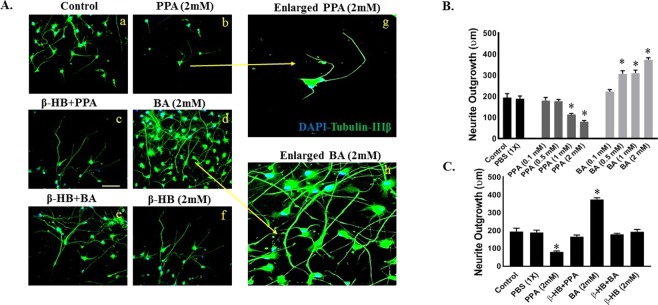
The Bivalent Role of PPA and BA on Neurite Growth *In Vitro*. Panel A(a–f) depicts double-immunostained representative images of differentiated neurons labeled with Tubulin-IIIβ and DAPI. Images (g,h) are enlarged views of (b,f), respectively. Magnification 25x and scale bar 25 um. Histogram B depict quantitative analysis of neurite outgrowth in (um) under control, PBS (1x), PPA (0.1, 0.5, 1, and 2 mM) and BA (0.1, 0.5, 1, and 2 mM). Histogram C depicts neurite outgrowth measurements in presence or absence of β-HB pre-treatment. Data is represented as Mean+SEM (n = 15 neurites per group) and statistical significance: (*p < 0.0001, F (10, 111) = 53.15) *versus* controls was obtained using Wilcoxon matched-pairs and One-way ANOVA followed by Tukey post-hoc test.

We evaluated how PPA and BA may affect neurite growth by measuring the longest neurite length of hNSC-derived neurons under ascending concentrations of PPA or PA. As shown in Fig. 7, PPA significantly decreased neurite length (Panel A-b and histogram 7B) to (115.2 um ± 4.4 um) at 1  and (80.70 um ± 5.5 um) at 2 mM compared to (194.93 um ± 19.07 um) in untreated cells. On the other hand, BA extended neurite growth with a minimum dose effect observed at 0.5 mM with neurite length reaching (308.30 um ± 13.9 um; Panel 7A-d and histogram B).

To test whether PPA or BA modulate neurite growth through GPR41 receptor, we measured neurite outgrowth in hNSCs pre-treated with β-HB. Clearly, β-HB blocked the effect of PPA and BA (Panel 7A-c and A-e). Specifically, β-HB pre-treatment resulted in neurite outgrowth measurements equivalent to controls (Fig. 7C). Statistical significance was ran using Wilcoxon matched-pairs and One-way ANOVA followed by Tukey post-hoc test). (*P < 0.0001, F (10, 111) = 53.15).

## Discussion

Gastrointestinal (GI) symptoms are among the most prevalent comorbidities associated with ASD^27^. A shift in gut microbiome and their by-products in autistic individuals has been reported^6–12,30^. Specifically, autistic gut seems to have an increase in *Clostridia spp*., *Bacteriodetes*, and *Desulfovibrio spp*. which are known to be active fermenters and producers of SCFAs including PPA and BA^7–12^. We were intrigued by MacFade et al finding that intracerebroventricular injection of PPA in rat’s brains induced reactive gliosis^11^. In the current study, we are linking maternal PPA exposure to disturbed neural patterning during early stages of embryonic neural development leading to over proliferation of glial cells, abnormal neural architecture, and increased inflammatory profile; possible precursors for autism. We employed a three-dimensional neurosphere assay to evaluate how SCFAs affect hNSC proliferation *in vitro*. Neurospheres are 3D progenitor cell conglomerates, representing a valuable *in vitro* model as they mirror the earliest stages of neural development^32^. They are useful to study cell proliferation, migration and differentiation making them a neurotoxic test of choice for plethora of agents and chemicals, particularly hormones, pesticides, or to study chemotherapy-induced neurotoxicity^33^. Our data unequivocally show that both PPA and BA promote hNSC self-renewal and proliferation *in vitro*, as evidenced by increased neurosphere number and diameter following exposure to either PPA or BA (Fig. 1). These results support earlier reports suggesting gut microbiota promote proliferation and maturation of enteric progenitor cells^34^. Our study provided additional evidence to support the proliferative role of gut by-products such as SCFAs on enteric progenitor cells. Furthermore, we demonstrated that such proliferative role is mediated through GPR41 receptor since its inactivation with β-HB voided SCFAs effect (Fig. 1).

Although disturbance in neuro/glia ratio in the autistic brain has been reported^21,22^, it was not clear how, when and why this dys-balance occurs. In this study, exposing differentiating hNSCs to 2 mM PPA induced a shift towards glial phenotype (Figs 2 and 3). This is an intriguing finding and a first in the field. Surprisingly, exposing differentiating hNSCs to BA favored the opposite, with increased neural proliferation (Figs 2 and 3). The SCFAs effect was confirmed following blocking GPR41 receptor with β-HB. This indicates that such effect is triggered by the specific binding of PPA and BA to GPR41 followed by a downstream molecular machinery leading to either glial or neural proliferation. Of importance here, the ratio of glia/neuron in BA treated cells is not as significant as that of PPA, possibly because PPA is the most potent activator of GPR41^29^. Over expression of *GPR41* in PPA-treated hNSCs confirmed differentiation shift to gliosis (Fig. 4).

PTEN was reported to regulate radial glia cell proliferation in the early stages of neural development through inhibition of Akt pro-survival pathway^26^. Recent studies reported that PTEN is downregulated in autistic glial cells^26,27^, however, what triggers PTEN inhibition in ASD remains uncertain. In this study, data suggest that PPA binding to its receptor may lead to GPR41-induced PTEN inhibition, thereof allowing Akt survival pathway to proceed. As we demonstrated in Fig. 5, PPA seems to tamper with both PTEN and activated p-Akt levels. *PTEN* expression decreased with increased PPA concentration and *vice versa* for p-Akt. Noteworthy, PPA interfered with the amount of activated p-Akt but not *Akt* expression. This result further validates that PPA has no direct effect on *Akt* expression but rather downregulates *PTEN* expression. Consequently, this allows p-Akt to remain active which results in over-proliferation of glia-committed neural progenitor cells.

To understand the inflammatory response and GI disorder in individuals with ASD, we studied the effect of PPA on gliosis and inflammatory cytokines in differentiated hNSCs. Our data showed that PPA seems to upregulate *TNF-α* and *IL-10* and increase the level of the cytokines (Fig. 6). Since PPA induced glial cell differentiation and increase in TNF-α and IL-10 transcription and translation, we propose that exposure to PPA during gestation may be related to gliosis and inflammation as reported in multiple neuro-developmental diseases including ASD. Specifically, exposure to high dose of PPA during early stages of neural stem cell development promotes proliferation and activation of glial cells, recapitulating the state of neuro-inflammation as reported in the post-partum autistic brain^18–20,35^.

In the developing brain, neurons are produced in the ventricular zone (VZ) and migrate into the developing neocortex guided by adjacent glial cells along the way^21,24^. Once settled, they undergo terminal differentiation in which long axons and dendrites extend to connect with adjacent neurons to form the final brain network, supported by glial cells^14,24^. It is therefore of outmost importance that the number and positioning of supporting glial cells be at chirurgical precision in order to achieve this delicate neuro-architecture. In the autistic brain, reports indicate that short and long distance inter-neuronal communication is disturbed, causing delays in information processing, increased repetitive behaviors and idiosyncrasies, as well as distortion in brain regions, such as the prefrontal cortex (PFC), associated with higher functioning^36^. However and as of latest data, it remained unclear what maybe causing neuronal circuitry disruption in ASD. We here stipulate that glial cells outnumbering neurons may constitute a physical barrier to the extending neurites, therefore accounting for a decrease in overall axonal growth. This phenomenon was clearly reflected in our data showing that PPA increased glial cell count and resulted in decreased neurite growth (Fig. 7). PPA may also block the molecular machinery involved in axonal expansion, nevertheless, further studies are needed in this area.

**Figure 8:**
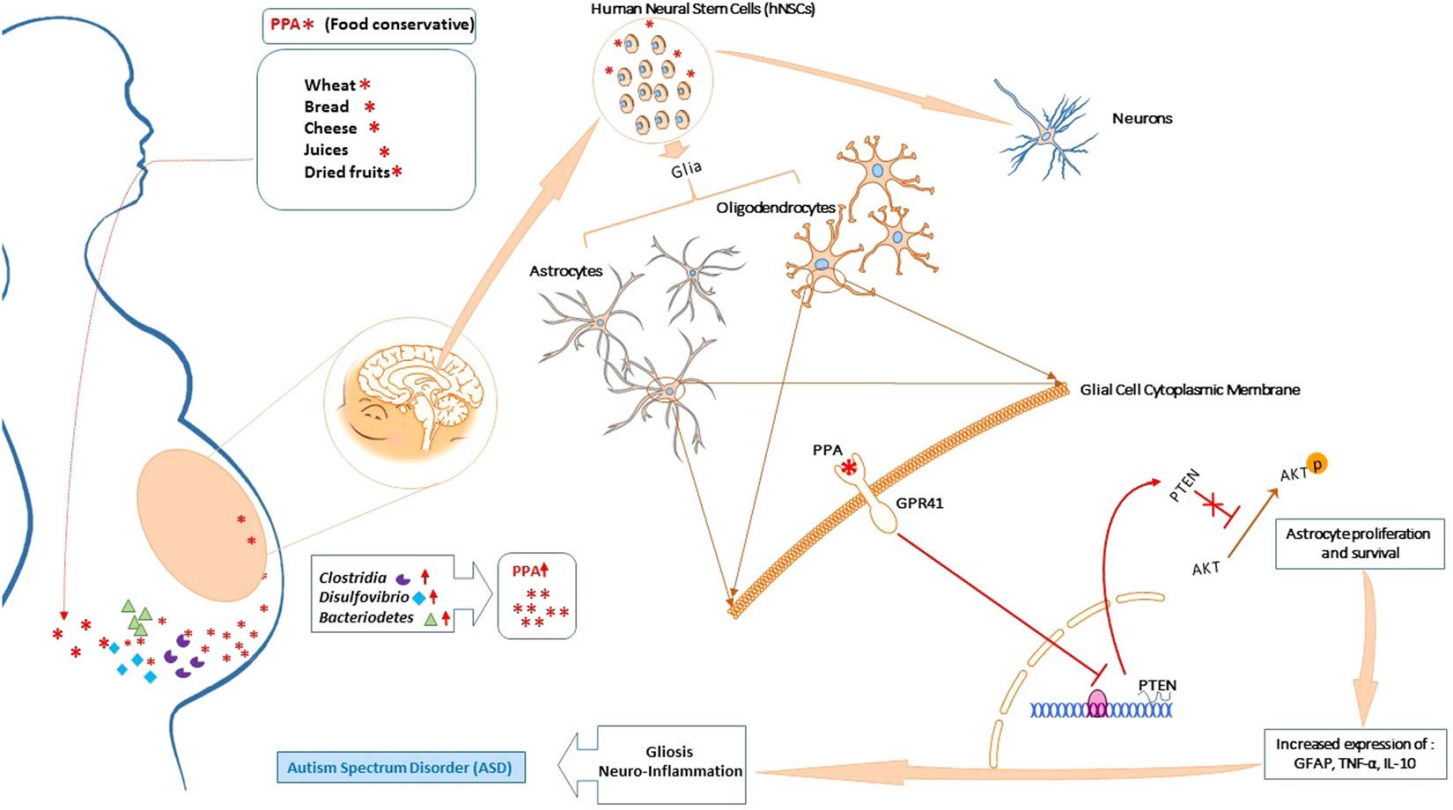
Overall Diagram for Hypothesized PPA Effect on hNSC *in vitro*. During the early stages of pregnancy, increased consumption of PPA-rich processed foods combined with pre-existent dysbiosis may lead to accumulation of PPA in the maternal GI, travel through general circulation, cross the placental barrier, and interfere with neural differentiation through binding to GPR41 receptor preferably expressed on glial progenitor cells. This will activate a downstream molecular pathway resulting in PTEN inhibition and activation of pro-survival Akt pathway, therefore favoring glial progenitor cells proliferation and differentiation. Mature glial cells will move on to produce inflammatory cytokines and release GFAP, all of which mimic gliosis and neuro-inflammation observed in ASD. Some illustrations used in this figure were originated from leased Motifolio (Scientific Illustration Toolkits for Presentations and Publications) materials.

Overall, the data in this study suggest that microbiome shift in maternal gut leads to formation of by-product such as PPA which then interferes with neural patterning during the early stages of the fetus’ neural development. This favors glial progenitor cells proliferation and survival leading to increased inflammatory profile and perturbed neural architecture. The data further suggests that such process is achieved through modulation of PTEN/Akt pathway within the growing glial cells but not neurons (Fig. 8).

Unexpectedly, BA, another SCFA used in this study as a control, seems to play a potential pro-neural role by virtue of increased neural cell count upon BA treatment (Figs 2 and 3). Interestingly, excess neural proliferation and macrocephaly were also linked to ASD^21,24,37^. However, what may be causing this shift remains largely debatable. We might be tempted to speculate that excess BA could be the culprit for the macrocephaly observed in ASD; however, more studies are warranted to make a better guided guess. Instead, it is safe assuming that the normal developing brain comes equipped with a set of neurons and supportive glial cells and any stirring away from this homeostatic ratio, towards glial overgrowth or neuronal over-proliferation, may disrupt the brain architecture potentially causing ASD. Therefore, and in light of these preliminary BA data, exploratory studies are more than warranted to elucidate BA role, if any, in ASD.

Solving the conundrical etiology of ASD is critical for any future prevention or treatment strategies. There is no doubt that genetic polymorphisms and environmental triggers are both involved in ASD development or at least in ASD complications. Because of the fact that autistic individuals who undergo antibiotic treatment seem to demonstrate a provisory yet noticeable relief from GI symptoms and some ASD behavior amelioration, and they may benefit from fecal replacement as a method to restore their microbiota pool^38,39^, there are good reasons to suggest that gut-brain axis is a potential culprit in ASD pathogenesis. This study is the first to link PPA and ASD-microbiome by-product to gliosis, disturbed neural architecture, and increase in inflammatory response, all of which may translate into dramatic neuro-complications including ASD.

## Data Availability

Raw data is available upon request.
